# Galectin-3 Negatively Regulates Hippocampus-Dependent Memory Formation through Inhibition of Integrin Signaling and Galectin-3 Phosphorylation

**DOI:** 10.3389/fnmol.2017.00217

**Published:** 2017-07-11

**Authors:** Yan-Chu Chen, Yun-Li Ma, Cheng-Hsiung Lin, Sin-Jhong Cheng, Wei-Lun Hsu, Eminy H.-Y. Lee

**Affiliations:** ^1^Graduate Institute of Life Sciences, National Defense Medical Center Taipei, Taiwan; ^2^Institute of Biomedical Sciences, Academia Sinica Taipei, Taiwan; ^3^Neuroscience Program in Academia Sinica Taipei, Taiwan

**Keywords:** galectin-3, integrin signaling, phosphorylation, contextual fear conditioning learning, spatial learning, hippocampus

## Abstract

Galectin-3, a member of the galectin protein family, has been found to regulate cell proliferation, inhibit apoptosis and promote inflammatory responses. Galectin-3 is also expressed in the adult rat hippocampus, but its role in learning and memory function is not known. Here, we found that contextual fear-conditioning training, spatial training or injection of NMDA into the rat CA1 area each dramatically decreased the level of endogenous galectin-3 expression. Overexpression of galectin-3 impaired fear memory, whereas galectin-3 knockout (KO) enhanced fear retention, spatial memory and hippocampal long-term potentiation. Galectin-3 was further found to associate with integrin α3, an association that was decreased after fear-conditioning training. Transfection of the rat CA1 area with small interfering RNA against galectin-3 facilitated fear memory and increased phosphorylated focal adhesion kinase (FAK) levels, effects that were blocked by co-transfection of the FAK phosphorylation-defective mutant Flag-FAKY397F. Notably, levels of serine-phosphorylated galectin-3 were decreased by fear conditioning training. In addition, blockade of galectin-3 phosphorylation at Ser-6 facilitated fear memory, whereas constitutive activation of galectin-3 at Ser-6 impaired fear memory. Interestingly galectin-1 plays a role in fear-memory formation similar to that of galectin-3. Collectively, our data provide the first demonstration that galectin-3 is a novel negative regulator of memory formation that exerts its effects through both extracellular and intracellular mechanisms.

## Introduction

Galectin-3 is a member of the galectin protein family—a large family of animal lectins whose members produce various biological effects (Barondes et al., 1994b) by interacting with other proteins through recognition of a β-galactoside conjugate on these proteins by the galectin carbohydrate-recognition domain (CRD; Barondes et al., 1994a). Because the amino acid sequences of the CRD in different galectins only share approximately 20%–40% homology (Oda et al., 1993), different galectins could interact with different glycoconjugated proteins, engaging different signaling pathways and yielding different biological effects, although some overlap in binding to the same glycoconjugates is also expected. Galectin-3 exists as a monomer in solution and forms pentamers upon binding to β-galactose on other proteins (Massa et al., 1993). This characteristic allows galectin-3 to function as a bridge among cells through its binding to multiple β-galactoses on different proteins.

Galectin-3, among the early-identified galectins, is known to regulate various cellular functions. For example, galectin-3 was found to enhance cell proliferation and inhibit apoptosis in different cell types in response to apoptotic stimuli (Liu et al., 2002; Hsu et al., 2006). Galectin-3 also plays a pro-inflammatory role (Rabinovich et al., 2007) and promotes tumor progression by modulating tumor cell survival and metastasis (Liu and Rabinovich, 2005). In addition, galectin-3 is involved in pre-mRNA splicing (Dagher et al., 1995) and cell-extracellular matrix (ECM) adhesion (Kuwabara and Liu, 1996).

These observations reveal a role for galectin-3 in various cells, but the role of galectin-3 in the nervous system has been less investigated. An earlier study reported the presence of galectin-3 in subsets of dorsal root ganglion and dorsal horn neurons in the rat spinal cord, suggesting a role in the development and function of sensory neurons (Regan et al., 1986). Another study showed that galectin-3 promotes oligodendrocyte differentiation and contributes to myelin integrity and function (Pasquini et al., 2011). Galectin-3 also plays a role in maintaining neuronal migration from the subventricular zone to the olfactory bulb in mice (Comte et al., 2011). Moreover, transient brain ischemia induces galectin-3 expression in microglial cells in the CA1 area of the gerbil hippocampus, a response that may contribute to the delayed neuronal death observed in this pathological setting (Satoh et al., 2011). However, the role of galectin-3 in learning and memory function has not been explored.

In the present study, we sought to determine whether, and how, galectin-3 regulates long-term memory formation. To address this, we adopted contextual fear-conditioning learning and water-maze learning as behavioral paradigms. Rats and galectin-3–knockout (KO) mice were used in conjunction with plasmid transfection and small interfering RNA (siRNA)-mediated knockdown approaches. Our results revealed that galectin-3 acts through inhibition of integrin signaling and galectin-3 phosphorylation to negatively regulate contextual fear memory and spatial memory formation.

## Materials and Methods

### Animals

Adult male Sprague-Dawley rats (250–350 g) and C57BL/6 mice were purchased from the BioLASCO and National Laboratory Animal Center, Taiwan, respectively. Galectin-3 KO (Gal-3^−/−^) mice were purchased from the Jackson Laboratory (strain name: B6.Cg-Lgals3^tm1Poi^/J, stock number: 006338). All animals were housed and maintained on a 12/12 h light/dark cycle (light on at 8:00 am) at the Animal Facility of the Institute of Biomedical Sciences (IBMS) with food and water continuously available. Only male mice were used in the present study. Experimental procedures followed the guidelines and ethical regulations of Animal Use and Care of the National Institute of Health and were approved by the Animal Committee of IBMS, Academia Sinica.

### Drug

N-methyl-D-aspartate (NMDA) was purchased from Tocris Bioscience (St. Louis, MO, USA) and was dissolved in PBS immediately before use.

### Hippocampal Cell Lysate Preparation

Animals were killed by decapitation, and their hippocampal tissue was dissected out. For most of the experiments, part of their dorsal CA1 tissue was further punched out for biochemical determinations. Rat hippocampal tissue was lysed by brief sonication in lysis buffer containing 50 mM Tris-HCl (pH 7.4), 150 mM NaCl, 2 mM EDTA, 1% IGEPAL CA-630, 1 mM phenylmethylsulfonyl fluoride (PMSF), 20 μg/ml pepstatin A, 20 μg/ml leupeptin, 20 μg/ml aprotinin, 50 mM NaF and 1 mM Na_3_VO_4_.

### Plasmid DNA Construction and Site-Directed Mutagenesis

For construction of the Flag-tagged galectin-3 plasmid, full-length *LGALS3* was cloned by amplifying the rat hippocampal cDNA with primers 5′-GGCGGATCCATGGCA GACGGCTTCTCACTTAATGATG-3′ (forward) and 5′-ATCAAG CTTTTAGATCATGGCGTGGGAAGCGCTGGTG-3′ (reverse). The PCR product was sub-cloned into the* BamHI* and *HindIII* sites of the mammalian expression vector pCMV-Tag2B (Invitrogen, Carlsbad, CA, USA). Flag-galectin-3S6A mutant plasmid was generated using the QuickChange Site-Directed Mutagenesis Kit (Stratagene, La Jolla, CA, USA). For construction of the Flag-focal adhesion kinase (FAK) vector, the previously constructed HA-FAK vector was used as a template (Yang et al., 2003) and was sub-cloned into the* BamHI* and *ApaI* sites of the pCMV-Tag2B expression vector with primers 5′-GGCGGATCC ATGGCAGCTGCTTATCTTGACCCAAAC-3′ (forward) and 5′-AAGGGCCCTCAGTGTGGCCGTGTCTGCCCTAGCATTT-3′ (reverse). Flag-FAKY397F was generated also using the QuickChange Site-Directed Mutagenesis Kit (Stratagene).

### Intra-Hippocampal Plasmid and siRNA Transfection and Drug Injection

Rats were anesthetized with pentobarbital (40 mg/kg) and subjected to stereotaxic surgery. Two 23-gauge, stainless steel, thin-walled cannulae were implanted bilaterally into the CA1 area of the rat brain at the following coordinates: 3.5 mm posterior to the Bregma, ±2.5 mm lateral to the midline, and 3.4 mm ventral to the skull surface. After recovery from surgery, N-methyl D-aspartate (NMDA; 12.5 mM) or phosphate-buffered saline (PBS) was directly injected into the CA1 area at a rate of 0.1 μl/min. A total of 0.7 μl was injected into each side. For transient transfection of galectin-3 and FAK expression plasmids, 0.7 μl of plasmid DNA complex (1.5 μg/μl) was bilaterally injected directly into the rat CA1 area using the non-viral cationic transfection agent, polyethyleneimine (PEI). For experiments that only required transient protein expression in the CA1 area, the method described in a previous study (Abdallah et al., 1996) was adopted. PEI was used because we have previously demonstrated that it does not exert toxicity towards hippocampal neurons (Chao et al., 2011). Before injection, plasmid DNA was diluted in 5% glucose to a stock concentration of 2.77 μg/μl. Branched, 25-kDa PEI (Sigma-Aldrich, St. Louis, MO, USA) was diluted to a concentration of 0.1 M in 5% glucose and added to the DNA solution. Immediately before injection, 0.1 M PEI was added to plasmid DNA to reach a PEI nitrogen/DNA phosphate ratio of 10, after which the mixture was vortexed for 30 s and allowed to equilibrate for 15 min. For *in vivo* transfections, 0.7 μl of galectin-3 siRNA (8 pmol) or control siRNA was bilaterally injected into the rat CA1 area, also using the transfection agent PEI. The sense and antisense sequences used for galectin-3 siRNA were 5′-GCAUGCUGAUCACAAUCAUdTdT-3′ and 5′- AUGAUUGUGAUCAGCAUGCdTdT-3′, respectively, whereas the corresponding sequences for galectin-1 siRNA were 5′-CUCAACAUGGAGGCCAUCAdTdT-3′ and 5′-UGAUGGCC UCCAUGUUGAGdTdT-3′. The sense and antisense sequences of Silencer Negative Control number 1 siRNA, used as a control, were 5′-UUCUCCGAACGUGUCACGUdTdT-3′ and 5′-ACGUGACACGUUCGGAGAAdTdT-3′, respectively. All siRNAs were synthesized by Ambion (Austin, TX, USA). For animals that received both galectin-3 siRNA and FAK plasmid transfections, siRNA and plasmids were mixed and injected together. The inner diameter of the injection needle was 0.31 mm, and the wall thickness of the needle was 0.12 mm. The needle was left in place for 5 min after injection to limit the diffusion of injected agent. Animals were sacrificed 15 min (for determination of phosphorylated extracellular signal-regulated kinase [pERK]) or 1 h (for galectin-1 and galectin-3 determination) after NMDA injection and 48 h after plasmid or siRNA transfection. Their brains were then removed and cut with a brain slicer. CA1 tissue containing the injection site was further punched out using a stainless steel, 2-mm-diameter punch as described previously (Chao et al., 2011). Tissues were frozen at −80°C until used for biochemical assays.

### Co-Immunoprecipitation (Co-IP)

The hippocampal tissue was lysed in RIPA buffer (50 mM Tris-HCl [pH 7.4], 150 mM NaCl, 1% IGEPAL CA-630, 1 mM EDTA and 1 mM EGTA) with cocktail of protease and phosphatase inhibitor (Roche, Penzberg, Germany). The clarified lysate (0.5 mg) was immunoprecipitated with 3 μl of anti-galectin-3 antibody (Catalog No. MAB1197, R&D systems, Minneapolis, MN, USA) or anti-integrin α3 antibody (Millipore, Bedford, MA, USA) at 4°C for 2 h. Twenty microliter (10% slurry) of the Mag Sepharose Xtra beads (GE Healthcare, Pittsburgh, PA, USA) was added to the IP reaction product for overnight to catch the immune complex. To confirm Flag-tagged plasmid transfection and expression in the CA1 area, the CA1 tissue was lysed in RIPA buffer and the clarified lysate (0.5 mg) was immunoprecipitated with 2 μl of Flag-M2 antibody at 4°C for 2 h. Twenty microliter (10% slurry) of the Mag Sepharose Xtra beads was similarly added to the IP product for overnight. The immune complex on beads were washed three times with PBS and boiled in sample buffer at 95°C for 10 min. The product was subjected to SDS-PAGE followed by transferring onto the PVDF membrane (Millipore) and immunoblotted with the indicated antibody.

### Western Blot

Cell lysates were resolved by 8%–12% SDS-PAGE and transferred onto the PVDF membrane. Immunoblotting was carried out using the following antibodies: rat anti-galectin-3 (1:5000, Catalog No. MAB1197, R&D systems, Minneapolis, MN, USA), goat anti-galectin-1 (1:5000, R&D systems), rabbit anti-FAK (1:5000), anti-phospho-FAK (Y397; 1:3000), anti-TNFα (1:3000), anti-IL6 (1:3000, Abcam, Cambridge, MA, USA), anti-integrin α3 (1:5000, Millipore), anti-NR1 (1:5000, Cell Signaling), mouse anti-galectin-3 (1:2000, Abcam), anti-actin (1:10,000, Millipore) and anti-Flag M2 (1:10,000, Sigma-Aldrich) antibodies. The secondary antibodies used were horseradish peroxidase (HRP)-conjugated goat-anti rabbit, goat-anti mouse, goat-anti rat and donkey-anti goat IgG antibodies (Jackson ImmunoResearch, West Grove, PA, USA). Membrane was developed by reacting with chemiluminescence HRP substrate (Millipore) and exposed to the LAS-3000 image system (Fujifilm, Tokyo, Japan) for visualization of protein bands. The protein bands were quantified using the NIH ImageJ Software.

### Immunohistochemistry

Rats were anesthetized with pentobarbital (40 mg/kg, i.p.) and perfused first with pre-chilled PBS and then with 4% paraformaldehyde. Brains were removed and post-fixed in a 20% sucrose/4% paraformaldehyde solution for 20–48 h. Frozen brains were cut into 30-μm sections on a cryostat and mounted on gelatin-coated slides. Brain sections were rinsed with PBS for 10 min, permeabilized by incubating with a pre-chilled EtOH/CH_3_COOH (95%:5%) solution for 10 min, washed three times with PBS, and pre-incubated in blocking solution (3% normal goat serum, 3% BSA and 0.2% Triton X-100 in PBS) for 2 h. For immunohistochemistry of galectin-3 in the mouse CA1 area, brain sections were incubated with goat anti-galectin-3 antibody (1:1000, Catalog No. AF1197; R&D Systems) at 4°C overnight. Brain sections were then washed with PBS and incubated with fluorescein isothiocyanate (FITC)-conjugated donkey anti-goat secondary antibody (1:500; Jackson ImmunoResearch, West Grove, PA, USA) for 1 h. For immunohistochemical detection of NeuN in the CA1 area, brain sections were incubated with mouse anti-NeuN antibody (1:1000; Millipore) at 4°C overnight. Brain sections were then washed with PBS and incubated with Cy3-conjugated goat anti-mouse secondary antibody (1:500; Jackson ImmunoResearch) for 1 h. For visualization of overexpressed Flag-galectin-3 in the CA1 area, brain sections were incubated with mouse anti-Flag M2 antibody (1:1000; Sigma-Aldrich) at 4°C overnight. Brain sections were then washed with PBS and incubated with Cy3-conjugated donkey anti-mouse antibody (1:500; Jackson ImmunoResearch) for 1 h. Sections were mounted on slides with VECTASHIELD mounting medium containing 4′,6-diamidino-2-phenylindole (DAPI; Vector Laboratories, Burlingame, CA, USA) at 4°C overnight. Photomicrographs were taken using a Zeiss LSM510 confocal microscope.

### Locomotor Activity Measurement

One day before measurement of locomotor activity, animals were habituated to an activity chamber (36 × 36 × 36 cm) for 10 min. The next day, they were placed in the same chamber, and locomotor activity was recorded for 15 min using a video camera. The chamber was divided into nine equal compartments. The number of crossovers between compartments, the total distance moved, the distance moved in the center region and movement speed were recorded and used as an index of locomotor activity.

### Contextual Fear-Conditioning Learning

One day before fear-conditioning learning, animals were habituated to the conditioning chamber (46 [L] × 30 [W] × 46 [H] cm; Med Associates Inc., Fairfax, VT, USA) for 10 min. Twenty-four hours later, animals were placed in the same chamber for contextual fear-conditioning training. After 3 min of free exploration, animals were trained with five electric foot shocks (0.8 mA, 1 s for mice; 1.2 mA, 1 s for rats), randomly administered over the subsequent 90 s, and their immediate freezing responses were recorded for the next 30 s. Twenty-four hours later, a retention test was performed for 5 min in the same chamber (without electric foot shock). The freezing response was measured and calculated as the percentage of time animals spent freezing during the recording period. For the non-trained control group, animals were also placed in the chamber for the same period of time as animals in the trained group, but no electric shock was delivered. Animals were sacrificed after the retention test or 24 h after training (or the non-trained control), and their brains were removed and cut by a brain slicer. Part of their dorsal CA1 tissue was further punched out by using a stainless steel, 2-mm-diameter punch, as shown in Supplementary Figure S1A. Tissues were frozen at −80°C until used for biochemical determinations.

### Shock Sensitivity Measurement

One day before the shock sensitivity test, animals were habituated to the chamber (46 [L] × 30 [W] × 46 [H] cm) for 10 min. Twenty-four hours later, they were placed in the same chamber for measurement of shock sensitivity. The protocol used was adopted from that of a previous study (Gulick and Gould, 2009). In brief, each animal was exposed to different intensities of foot shocks (0.1–1.0 mA) for 2 s for each stimulus, with an 18-s inter-stimulus interval and a 90-s inter-trial interval. Their motion was scored (0, no response; 1, hop; 2, jump; 3, run; 4, horizontal jump; 5, vertical jump) and the number of vocalizations in response to each shock was measured.

### Water Maze Learning

The water maze used for rats was a plastic, circular pool with 2.0 m in diameter and 0.6 m in height and filled with water (25 ± 2°C, water was made cloudy by adding milk powder) to a depth about 20 cm. A circular platform (13 cm in diameter) was placed at a specific location away from the edge of the pool and submerged 1.5 cm below the water surface. Distinctive, visual cues were set on the wall. For spatial training, naïve rats were randomly divided to two groups (non-trained and trained) and were subjected to water-maze learning for three trials a day with a total of 15 trials (in 5 days). For each trial, animals were placed at three different starting positions of the pool. Animals were given 60 s to find the platform. If an animal could not find the platform, it was guided to the platform and allowed to stay on it for 20 s. To test their memory retention, a probe trial of 60 s was given the next day after the end of 5-day training. For the probe trial test, animals were placed in the pool with the platform removed and the time they spent in each quadrant (target quadrant (T), left quadrant (L), opposite quadrant (O), and right quadrant (R)) and their swimming paths were recorded. For the water maze training experiment, animals in the swim control group (non-trained group) swam for the average time in each trial as that in the trained group except that the platform and visual cues were not present. Animals were sacrificed after the probe trial test or at the end of training (or swim control) and part of their dorsal CA1 tissue was punched out for biochemical assays as described above (also shown in Supplementary Figure S1A).

For visible platform learning, a flag was mounted on the platform and the platform was raised 2.5 cm above the surface of water. In addition, milk powder was not added so the animals could see the location of the platform from the water.

### Extracellular Field Potential Recording

Wild-type and galectin-3 KO mice were used for electrophysiological recording. Animals were sacrificed and their brain slices were transferred to an immersion-type recording chamber, perfused with ACSF containing 100 μM picrotoxin at a rate of 2 ml/min at room temperature. An incision was made between the CA1 and CA3 areas to remove afferent input from CA3. For the extracellular field potential recording, a glass pipette filled with 3 M NaCl was positioned in the CA1 stratum radiatum area to record fEPSP. Bipolar stainless steel stimulating electrodes (Frederick Haer Company, Bowdoin, ME, USA) were placed in the striatum radiatum to stimulate the Schaffer collateral pathway. Stable baseline fEPSP activity was recorded by applying a short-duration current stimulation pulse (~40 μs) at a predetermined intensity every 15 s for at least 20 min. LTP was induced by using the TBS paradigm according to that described previously (Tai et al., 2016). Briefly, three trains of theta-burst stimulation were delivered. Each train consisted of 10 sets of bursts (4 stimuli, 100 Hz) with an inter-burst interval of 200 ms. The interval between each stimulus train was 20 s. The method used to obtain the input/output response was adopted from that of a previous study (Lemtiri-Chlieh et al., 2011). fEPSPs were evoked at 0.1 Hz by stimulation of the Schaffer collaterals with a bipolar steel stimulating electrode (10 MΩ; FHC) placed in the hippocampal CA1 stratum radiatum and fEPSPs were recorded at room temperature from the CA1 layer using borosilicate glass microelectrodes (8–10 MΩ) filled with 3 M NaCl. To obtain an input/output relationship curve, afferent Schaffer collaterals were stimulated at increasing stimulus amplitudes (typically constant current pulses at 5, 10, 15, 20, 25, 30, 35, 40, 45, 50, 60, 70, 80, 90, 100, 110 and 120 μA). The initial slope of the fEPSP was measured from linear regression using Signal 4.11 Software.

### Statistics

All data are presented as mean values ± SEM. Biochemical and fear conditioning data were analyzed by Student’s *t*-test or by one-way analysis of variance (ANOVA) followed by Newman-Keuls multiple comparisons (represented by the *q* value). Acquisition data from water maze learning were analyzed by two-way repeated ANOVA with trial as the repeated measure and group as the independent variable. The Newman-Keuls multiple comparison statistics was further used to test the difference between the WT and galectin-3 KO groups (represented by the *q* value). The probe trial data were analyzed with the Chi-square analysis with 25% as the expected frequency (four quadrants in all), followed by *t*-test to compare the difference between the WT and galectin-3 KO group. Values of *P* < 0.05 were considered statistically significant (*P < 0.05, **P < 0.01, ^#^*P* < 0.001).

## Results

### Regional Distribution of Galectins in the Rat Brain and Endogenous Galectin-3 Expression in the Mouse CA1 Area

**Figure 1:**
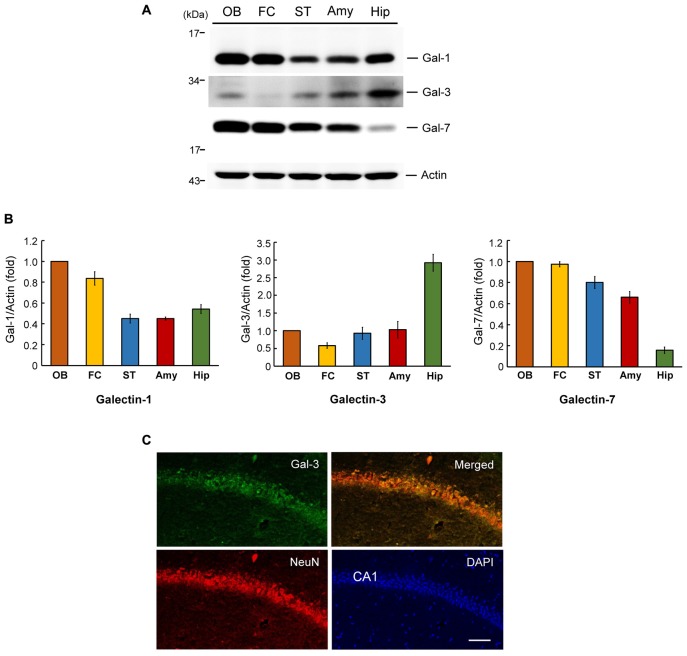
Regional distribution of galectins in the rat brain and endogenous galectin-3 expression in the mouse CA1 area. **(A)** Western blot analysis showing the expression level of galectin-1, galectin-3 and galectin-7 in several regions in the rat brain. **(B)** Quantitative analyses of galectin-1, galectin-3 and galectin-7 expression level in different brain regions. *N* = 4 each group. Gal-1: galectin-1, Gal-3: galectin-3, Gal-7: galectin-7. OB: olfactory bulb, FC: frontal cortex, ST: striatum, Amy: amygdale, Hip: hippocampus. Data are mean ± SEM. **(C)** Immunohistochemistry showing the distribution of galectin-3, NeuN and their co-localization in rat hippocampal CA1 area. Scale bar equals 50 μm.

We first examined the expression level of galectin-3 in several brain regions in the rat that have been implicated in neuronal plasticity or development. These analyses indicated that the expression level of galectin-3 is approximately 2.5–6-fold higher in the hippocampus than in other areas examined, including the frontal cortex, olfactory bulb, striatum and amygdala (Figures 1A,B, middle panel). This expression pattern implies that galectin-3 may play a role in regulating learning and memory function. We also examined the expression pattern of two other galectin family proteins, galectin-1 and galectin-7, in the same brain regions of these animals. This examination revealed a maximum difference in galectin-1 expression level of approximately 2.5-fold among the brain areas examined, with the olfactory bulb showing the highest expression (Figures 1A,B, left panel). Galectin-7 expression was more evenly distributed among most of these areas, except that its expression level was much lower (~4–6-fold) in the hippocampus than in other brain areas examined (Figures 1A,B, right panel). This differential expression pattern suggests that different galectins may be involved in different brain functions.

To further assess endogenous galectin-3 expression in the hippocampal CA1 area, we performed an immunohistochemical analysis of galectin-3 expression in normal mouse brain tissue. In these experiments, tissue was also immunostained for NeuN, a neuronal marker and counterstained with the nuclear dye, DAPI. These analyses revealed that galectin-3 was expressed in the CA1 layer (green), as was NeuN (red), although galectin-3 staining intensity was not high. Moreover, merged images showed that galectin-3 expression overlapped with that of NeuN (Figure 1C). No specific labeling was observed in WT mice incubated with the FITC-conjugated secondary antibody only (Supplementary Figure S1B). Galectin-3 expression was not observed in galectin-3 KO mice either (Supplementary Figure S1C). Both of them showed background staining only.

### Contextual Fear-Conditioning Training and NMDA Treatment Decrease Galectin-3 Expression in the Rat Hippocampus

**Figure 2:**
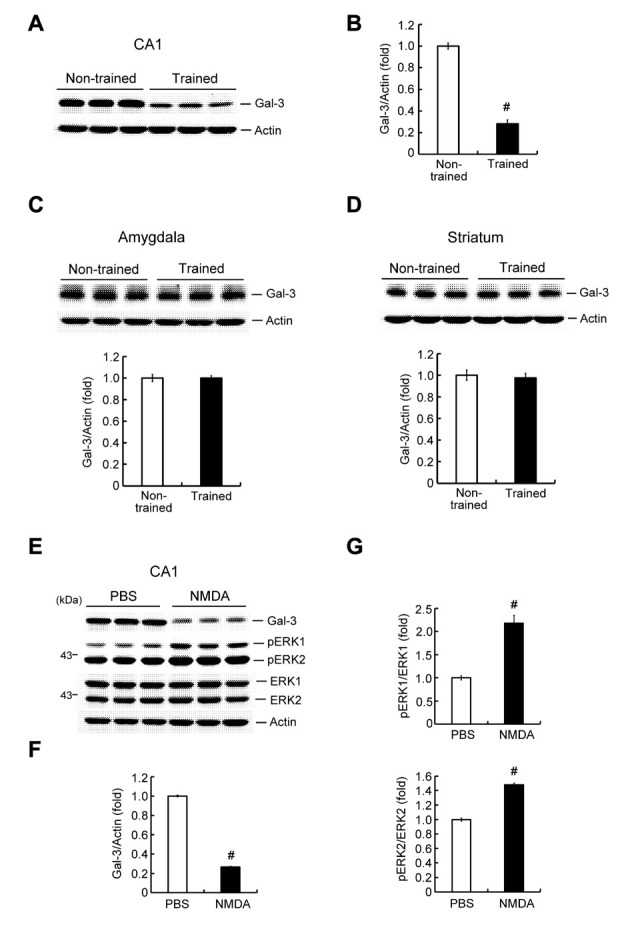
Contextual fear-conditioning training and NMDA decrease galectin-3 expression in the rat hippocampus. **(A)** Western blot showing galectin-3 expression in the CA1 area of trained and non-trained rats. **(B)** Quantitative analysis of galectin-3 expression in CA1 area of trained and non-trained rats (*t*_(1,18)_ = 15.27, *P* < 0.001). **(C)** Western blot and quantitative analysis of galectin-3 expression in the amygdala of trained and non-trained rats (*t*_(1,18)_ = 0.02, *P* > 0.05). **(D)** Western blot and quantitative analysis of galectin-3 expression in the striatum of trained and non-trained rats (*t*_(1,18)_ = 0.38, *P* > 0.05). *N* = 10 each group. **(E)** Western blot showing the effect of NMDA injection (12.5 mM) on the expression level of galectin-3 and pERK in rat CA1 area. Quantitative analysis of the effect of NMDA on **(F)** Galectin-3 expression (*t*_(1,10)_ = 51.11, *P* < 0.001) and **(G)** pERK1/ERK1, pERK2/ERK2 levels (*t*_(1,10)_ = 6.69, *P* < 0.001 for pERK1 and *t*_(1,10)_ = 15.12, *P* < 0.001 for pERK2) in the CA1 area. *N* = 6 each group. Data are mean ± SEM. ^#^*P* < 0.001.

To study the possible involvement of galectin-3 in learning and memory formation, we first examined whether learning alters the endogenous expression level of glaectin-3 in the hippocampus. Rats were randomly divided to two groups. One group was subjected to contextual fear conditioning training; the other served as non-trained controls. For the non-trained group, animals were placed in the same chamber, but no shock was delivered. Animals were sacrificed 1 h after measuring immediate freezing behavior, after which their brains were collected and hippocampal CA1 tissue was dissected out and analyzed for galectin-3 expression by Western blotting. These analyses revealed that contextual fear-conditioning training decreased galectin-3 expression by approximately 3.5-fold in the rat CA1 area (Figures 2A,B), but did not alter galectin-3 expression in the amygdala (Figure 2C) or striatum (Figure 2D). The amygdala has also been implicated in memory of associative fear conditioning; thus, galectin-3 expression might also be altered in this area at a different time point after training. To confirm that the above alteration of hippocampal galectin-3 expression was indeed caused by training-induced neuronal activation, we further examined the effect of NMDA injection (12.5 mM) into the rat CA1 area on galectin-3 expression; control animals received an injection of PBS. Animals were sacrificed 1 h after NMDA (or PBS) injection, and their CA1 tissue was analyzed for galectin-3 expression by Western blotting. A separate group of animals, used for determination of pERK1 and pERK2 levels, was sacrificed 15 min after NMDA injection. These analyses revealed that NMDA produced nearly a 4-fold decrease in galectin-3 expression in the CA1 area (Figures 2E,F). pERK1 and pERK2 levels in the CA1 area were also markedly increased, confirming the neuronal-activation effect of NMDA injection (Figures 2E,G).

### Overexpression of Galectin-3 Impairs Fear Memory, Whereas Galectin-3 Knockout Enhances Fear Memory and Long-Term Potentiation

**Figure 3:**
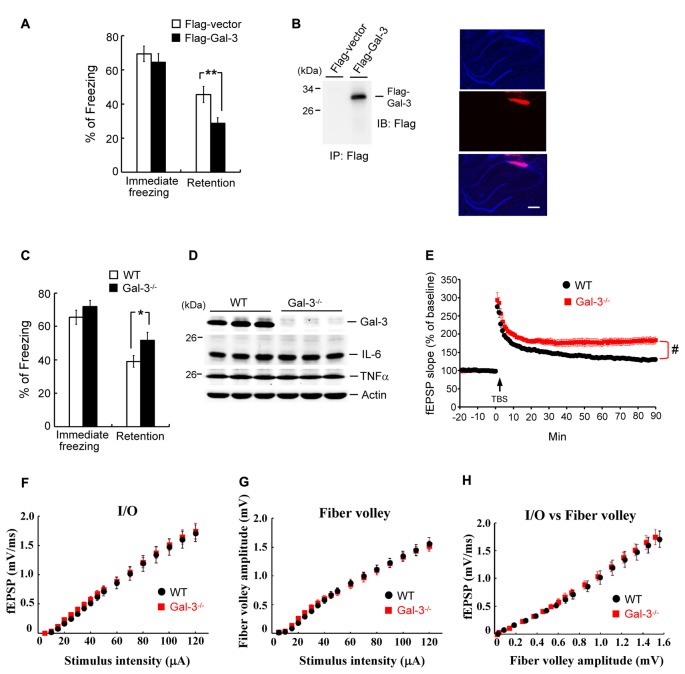
Overexpressionof galectin-3 impairs fear memory but fear memory is enhanced in galectin-3 knockout (KO) mice. **(A)** Immediate freezing response (*t*_(1,14)_ = 0.74, *P* > 0.05) and retention performance (*t*_(1,14)_ = 2.95, *P* = 0.01) in rats transfected with Flag-galectin-3WT plasmid or Flag-vector plasmid. *N* = 8 each group. **(B)** Immunoprecipitation and immunoblotting against the Flag tag confirming the expression of galectin-3 in Flag-galectin-3 transfected animals (left). Immunohistochemistry with Cy3-conjugated secondary antibody against Flag confirming the expression of galectin-3 in rat CA1 area (right). Scale bar equals 250 μm. **(C)** Immediate freezing response (*t*_(1,18)_ = 1.1, *P* > 0.05) and retention performance (*t*_(1,18)_ = 2.14, *P* < 0.05) in wild-type (WT) and galectin-3 KO (Gal-3^−/−^) mice. *N* = 10 each group. **(D)** Western blot showing the expression level of galectin-3, IL-6 and TNFα in WT and Gal-3^−/−^ mice (*t*_(1,18)_ = 0.4 for IL-6 and *t*_(1,18)_ = 0.54 for TNFα, both *P* > 0.05). *N* = 10 each group. **(E)** fEPSP slope in WT and galectin-3 KO mice subjected to TBS stimulation and LTP recording (*t*_(1,14)_ = 4.32, *P* < 0.001). *N* = 8 each group. TBS: theta burst stimulation. **(F)** The input/output (I/O) fEPSP slope (*F*_(1,32)_ = 0.045, *P* > 0.05), **(G)** the amplitude of fiber volley (*F*_(1,32)_ = 0.004, *P* > 0.05) and **(H)** the I/O vs. fiber volley curve (*F*_(1,32)_ = 0.36, *P* > 0.05) were generated in response to different intensities of presynaptic fiber stimulation of the Schaffer collateral pathway. *N* = 8 each group. Data are mean ± SEM. *P < 0.05, **P < 0.01, ^#^*P* < 0.001.

The above results indicate a negative correlation between learning and galectin-3 expression in the hippocampus. Here, we examined the causal relationship between hippocampal galectin-3 expression and learning. Rats were randomly divided into two groups. In one group, the CA1 area was transfected with a Flag-galectin-3 expression plasmid; in the other, a Flag-vector (control) was administered. Rats were subjected to contextual fear-conditioning training 24 h after transfection, and fear retention was measured 24 h after training. These experiments revealed that the immediate freezing response was similar between these two groups of rats, but Flag-galectin-3 transfection markedly impaired fear retention (Figure 3A). To confirm overexpression of galectin-3 in Flag-galectin-3–transfected animals, we immunoprecipitated their CA1 tissue lysate and immunoblotted it with an anti-Flag antibody. A Flag-galectin-3 band was observed only in the overexpression group (Figure 3B, left panel). We further confirmed galectin-3 overexpression by immunohistochemistry using a Cy3-conjugated secondary antibody against Flag. Red Cy3 fluorescence was detected in the CA1 area, where it was co-localized with DAPI staining (Figure 3B, right panel).

If overexpression of galectin-3 impairs fear memory, knockdown of galectin-3 would be expected to facilitate fear memory. To test this, we subjected wild-type (WT) and galectin-3–KO mice to contextual fear-conditioning training, and measured fear retention 24 h later. These experiments revealed that, although the immediate freezing response was similar between these two groups of mice, retention performance was better in galectin-3–KO mice than in WT controls (Figure 3C). A subsequent Western blot analysis confirmed the lack of galectin-3 expression in galectin-3–KO mice (Figure 3D). Because galectin-3 is involved in inflammation, we also examined whether enhanced fear retention in galectin-3–KO mice is attributable to altered immune responses in these animals. Interleukin-6 (IL-6) and tumor necrosis factor-alpha (TNFα) are expressed in microglia cells and astrocytes in the brain, and their expression level is increased upon inflammation and brain damage (Lucas et al., 2006; Woodcock and Morganti-Kossmann, 2013); therefore, we determined IL-6 and TNFα levels in the CA1 area of these animals. These experiments showed that IL-6 and TNFα expression levels were similar between WT and galectin-3–KO mice (Figure 3D), indicating that the altered fear memory in galectin-3–KO mice was not attributable to inflammation in the brains of these animals.

We further examined the role of galectin-3 in neuronal plasticity using the long-term potentiation (LTP) paradigm. Both WT and galectin-3–KO mice were subjected to tetanic stimulation, and field potentials were recorded. These experiments revealed that theta burst stimulation produced a persistent increase in the slope of field excitatory postsynaptic potentials (fEPSPs) in CA1 neurons of WT mice (~130%–160% of baseline), an effect that was further enhanced in galectin-3–KO mice (~180% of baseline; Figure 3E). We further examined whether galectin-3–KO mice exhibit altered basal synaptic transmission, which could contribute to the observed enhancement of LTP. The input/output (I/O) fEPSP slope and fiber volley amplitude produced by different intensities of presynaptic fiber stimulation of the Schaffer collateral pathway were recorded, and an I/O vs. fiber volley curve was generated. These experiments revealed no differences in fEPSP slope, fiber volley amplitude, or I/O curve between WT and galectin-3–KO mice (Figures 3F–H).

### Water-Maze Training Decreases Galectin-3 Expression in the Rat Hippocampus

**Figure 4:**
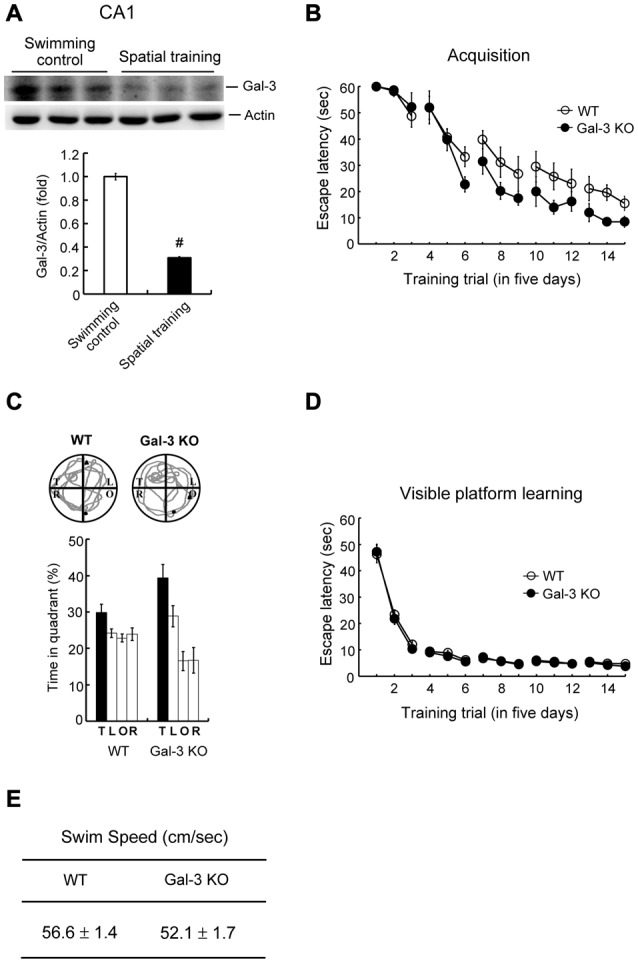
Water-maze training decreases galectin-3 expression in the rat hippocampus and spatial memory is enhanced in galectin-3 KO mice. **(A)** Western blot showing galectin-3 expression in CA1 area of rats subjected to water-maze training or swimming control. The quantified result is also shown (*t*_(1,10)_ = 23.75, *P* < 0.001). *N* = 6 each group. **(B)** Spatial acquisition of WT and galectin-3 KO mice (*F*_(1,14)_ = 5.34, *q* = 3.33, *P* < 0.05). **(C)** Representative swim pattern and probe trial performance of WT and galectin-3 KO mice (Chi square followed by *t*-test, *t*_(1,14)_ = 1.61, *P* > 0.05). *N* = 8 each group. **(D)** Visible platform learning of WT and galectin-3 KO mice (*F*_(1,14)_ = 0.56, *P* > 0.05). **(E)** The swim speed of WT and galectin-3 KO mice (*t*_(1,14)_ = 2.06, *P* > 0.05). *N* = 8 each group. Data are mean ± SEM. ^#^*P* < 0.001.

To understand whether galectin-3 is also regulated by other hippocampus-dependent learning tasks, we examined the effect of spatial training on galectin-3 expression. Animals were randomly divided into two groups; one was subjected to water-maze training, and the other served as swimming controls. At the end of training (or swimming), rats in both groups were sacrificed, and part of their dorsal CA1 tissue was punched out and subjected to Western blot analysis of galectin-3 expression. These analyses revealed that water-maze training decreased galectin-3 expression by approximately 3.5-fold (Figure 4A). Next, we compared performance of WT and galectin-3–KO mice in water-maze learning and probe trial tests. These experiments showed that spatial acquisition was enhanced in galectin-3–KO mice compared with WT controls (Figure 4B). Galectin-3–KO mice also spent more time in the target quadrant in the probe trial test, but the difference with WT animals is not statistically significant (Figure 4C).

We next examined whether there are alterations in sensorimotor function in galectin-3–KO mice that might consequently affect their spatial learning and memory performance. WT and galectin-3–KO mice subjected to visible platform learning for the same number of trials performed similarly in visible platform learning (Figure 4D) and also exhibited similar swim speeds (Figure 4E).

### Galectin-3 Negatively Regulates Fear Memory through Inhibition of Integrin α3-Mediated Signaling

**Figure 5:**
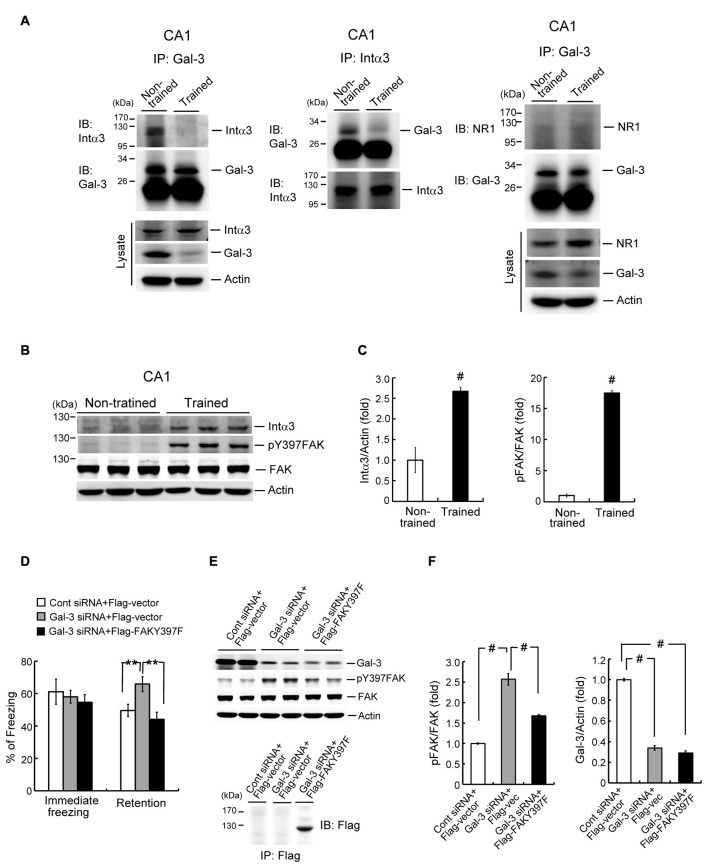
Galectin-3 negatively regulates fear memory through inhibition of integrin α3-mediated signaling. **(A)** Left and middle panel, Co-IP experiment showing galectin-3 association with integrin α3 and *vice versa* in rat CA1 area. This association is reduced after training (upper panels). Western blot showing the expression of integrin α3 and galectin-3 in trained and non-trained animals (lower-left panel). Right panel, Co-IP experiment showing that galectin-3 is not associated with NR1 in rat hippocampus, and fear-conditioning training does not apparently alter this relationship. Western blot showing that NR1 expression is increased, but galectin-3 expression is decreased in hippocampal cell lysate of trained animals (lower panel). Experiments are in two repeats. **(B)** Western blot showing the expression level of integrin α3 (*t*_(1,10)_ = 5.18, *P* < 0.001), phospho-focal adhesion kinase (FAK; *t*_(1,10)_ = 36, *P* < 0.001) and FAK in trained and non-trained animals. **(C)** Quantitative analysis of integrin α3 and phospho-FAK expression in trained and non-trained animals. *N* = 6 each group. **(D)** Immediate freezing response (*F*_(2,24)_ = 0.33, *P* > 0.05) and retention performance in galectin-3 siRNA + Flag-vector and galectin-3 siRNA + Flag-FAKY397F co-transfected animals (*F*_(2,24)_ = 7.0, *P* < 0.001; *q* = 3.81, *P* = 0.01 comparing the galectin-3 siRNA + Flag-vector group with control siRNA + Flag-vector group; *q* = 5.08, *P* < 0.01 comparing the galectin-3 siRNA + Flag-FAKY397F group with galectin-3 siRNA + Flag-vector group). *N* = 9 each group. **(E)** Western blot showing the expression level of galectin-3 (*F*_(2,24)_ = 476.38, *P* < 0.001; *q* = 36.42 and *q* = 39.05, respectively; both *P* < 0.001), phospho-FAK (*F*_(2,24)_ = 87.36, *P* < 0.001; *q* = 18.63, *P* < 0.001 comparing the galectin-3 siRNA + Flag-vector group with control siRNA + Flag-vector group; *q* = 10.63, *P* < 0.001 comparing the galectin-3 siRNA + Flag-FAKY397F group with galectin-3 siRNA + Flag-vector group) and FAK in galectin-3 siRNA + Flag-vector and galectin-3 siRNA + Flag-FAKY397F co-transfected animals. *N* = 9 each group. Immunoprecipitation and immunoblotting against Flag confirming the expression of FAKY397F in Flag-FAKY397F-transfected animals (lower panel). **(F)** Quantitative analysis of galectin-3, phospho-FAK and FAK expression in the same animals. *N* = 9 each group. Data are mean ± SEM. **P < 0.01, ^#^*P* < 0.001.

After establishing the role of galectin-3 in negative regulation of fear memory and spatial memory formation, we next examined its underlying mechanism. It has been suggested that galectin-3 binds to integrin through binding of its CRD to β-galactose-conjugated integrin (Hughes, 2001; Ochieng et al., 2002). Moreover, integrin has been shown to play a facilitating role in learning and memory function in both *Drosophila* and mice (Grotewiel et al., 1998; Chan et al., 2003). Collectively, these observations suggest the possibility that galectin-3 negatively regulates memory through inhibition of integrin-mediated signaling. Integrins are composed of α and β subunits (Ruoslahti and Pierschbacher, 1987; Humphries et al., 2006), and integrin α3β1 has been shown to mediate neurite outgrowth (DeFreitas et al., 1995). Thus, to test the role of integrins in galectin-3 effects on memory, we examined the association between galectin-3 and integrin α3 in the rat hippocampus, and determined whether contextual fear conditioning training alters this association. Co-immunoprecipitation experiments in which cell lysates were immunoprecipitated with an anti-galectin-3 antibody and immunoblotted with an anti-integrin α3 antibody revealed that galectin-3 was associated with integrin α3 in the CA1 area of non-trained rats. Notably, this association was dramatically decreased in rats subjected to fear-conditioning training (Figure 5A, left panel). Similar results were obtained by immunoprecipitating cell lysates with an anti-integrin α3 antibody and immunoblotting with an anti-galectin-3 antibody (Figure 5A, middle panel). Increased integrin α3 expression and decreased galectin-3 expression were also observed in cell lysates of trained animals compared with non-trained controls (Figure 5A, lower-left panel). These results confirm the effects of training.

Because the NMDA receptor (NMDAR) plays a key role in mammalian learning and memory function, and NR1 is an essential subunit of the NMDAR (Izquierdo, 1991; Sheng et al., 1994), we also examined the association between galectin-3 and NR1 in the hippocampus of non-trained and trained rats. These analyses revealed no association between galectin-3 and NR1 in non-trained animals, and fear-conditioning training did not appear to alter this relationship (Figure 5A, right panel). There was, however, an increase in the expression level of NR1 in cell lysates of trained animals compared with non-trained controls, but the expression level of galectin-3 was decreased (Figure 5A, lower-right panel). These results confirm the effect of training.

FAK activation has also been suggested to mediate integrin signaling and integrin-mediated neuronal function (Guan, 1997; Robles and Gomez, 2006). Given that integrin-mediated signaling facilitates learning and memory, training would be expected to activate FAK and perhaps increase the expression of integrin as well. To examine this issue, we divided rats into two groups, and subjected one group to contextual fear-conditioning training; the other group served as non-trained controls. Rats were sacrificed 1 h after measuring immediate freezing responses, and their CA1 tissue was analyzed by Western blotting. These analyses revealed that fear-conditioning training markedly increased the expression level of integrin α3. It also increased the level of Tyr-397–phosphorylated FAK, without affecting total FAK expression levels (Figures 5B,C).

Because overexpression of galectin-3 impaired fear memory, and galectin-3 interactions with integrin α3 were reduced after fear conditioning learning, the prediction is that knockdown of galectin-3 would enhance fear memory through disinhibition of integrin α3-mediated signaling. This hypothesis was examined by dividing animals randomly into three groups and transfecting their CA1 regions with the following plasmids: control siRNA + Flag-vector, galectin-3 siRNA + Flag-vector, or galectin-3 siRNA + Flag-FAKY397F. Rats were subjected to contextual fear-conditioning learning and memory tests 24 h and 48 h after plasmid transfection, respectively. After the fear-memory test, rats were sacrificed and their CA1 tissue was analyzed by Western blotting. These analyses revealed that immediate freezing responses were similar among these three groups of rats. Transfection of galectin-3 siRNA markedly enhanced fear retention, an effect that was blocked by co-transfection of Flag-FAKY397F (Figure 5D). Western blot analyses showed that galectin-3 siRNA transfection significantly increased the level of Tyr-397–phosphorylated FAK, an effect that was similarly blocked by co-transfection of Flag-FAKY397F (Figures 5E,F, left panel). Concurrent with this, galectin-3 expression level was markedly decreased in both galectin-3 siRNA-transfected groups (Figures 5E,F, right panel). Flag-FAKY397F plasmid transfection and expression were confirmed by immunoprecipitation and immunoblotting using an anti-Flag antibody (Figure 5E, lower panel).

### Galectin-3 Negatively Regulates Fear Memory through Phosphorylation of Galectin-3 at Ser-6

**Figure 6:**
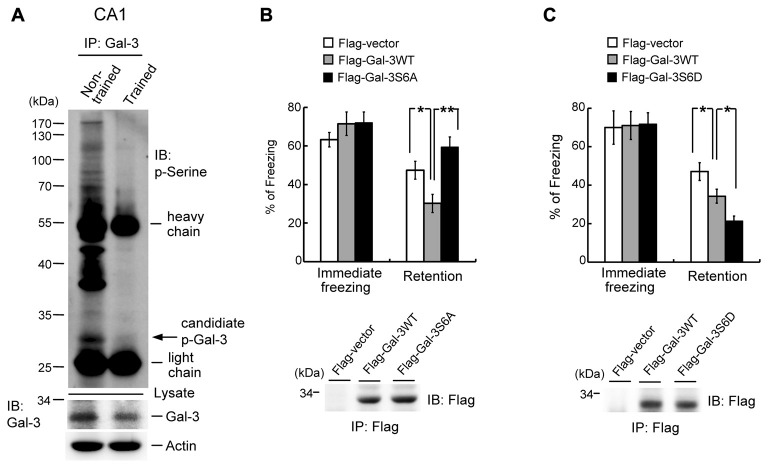
Galectin-3 negatively regulates fear memory through phosphorylation of galectin-3 at Ser-6. **(A)** Co-IP experiment (immunoprecipitation with anti-galectin-3 antibody and immunoblotting with anti-p-Ser antibody) showing the candidate p-galectin-3 band in non-trained animals in rat CA1 area. This association is dramatically reduced after training (upper panel). Western blot showing the expression level of galectin-3 in trained and non-trained animals (lower panel). Experiments are in two repeats. **(B)** Immediate freezing response (*F*_(2,21)_ = 0.88, *P* > 0.05) and retention performance in galectin-3WT and galectin-3S6A-transfected animals (*F*_(2,21)_ = 8.89, *P* < 0.001; *q* = 3.49, *P* < 0.05 comparing the galectin-3WT with Flag-vector group; *q* = 5.93, *P* = 0.01 comparing the Flag-galectin-3S6A group with Flag-galectin-3WT group; *q* = 2.44, *P* > 0.05 comparing the Flag-galectin-3S6A group with Flag-vector group). *N* = 8 each group. Immunoprecipitation and immunoblotting against the Flag tag confirming the expression of galectin-3 and galectin-3S6A in Flag-galectin-3WT and Flag-galectin-3S6A-transfected animals, respectively (lower panel). **(C)** Immediate freezing response (*F*_(2,21)_ = 0.01, *P* > 0.05) and retention performance in galectin-3WT and galectin-3S6D-transfected animals (*F*_(2,21)_ = 11.86, *P* < 0.001; *q* = 3.42, *P* < 0.05 comparing the galectin-3WT with Flag-vector group; *q* = 3.47, *P* < 0.05 comparing the Flag-galectin-3S6D group with Flag-galectin-3WT group; *q* = 6.89, *P* < 0.001 comparing the Flag-galectin-3S6D group with Flag-vector group). *N* = 8 each group. Immunoprecipitation and immunoblotting against the Flag tag confirming the expression of galectin-3 and galectin-3S6D in Flag-galectin-3WT and Flag-galectin-3S6D-transfected animals, respectively (lower panel). Data are mean ± SEM. *P < 0.05, **P < 0.01.

The finding that galectin-3 negatively regulates fear memory through inhibition of integrin α3-mediated signaling indicates an extracellular role of galectin-3. Galectin-3 has also been shown to exert intracellular functions, and phosphorylation of galectin-3 at Ser-6 and Ser-12 by casein kinase I is one mechanism through which such functions are mediated (Huflejt et al., 1993). Accordingly, we examined whether intracellular galectin-3 also negatively regulates fear memory through such a mechanism by studying the effects of galectin-3 phosphorylation. Because no specific antibodies against p-Ser-6 galectin-3 are commercially available, we first examined whether the level of Ser-phosphorylated galectin-3 is altered by fear conditioning training. Accordingly, rats were divided into two groups; one was subjected to fear-conditioning training, and the other served as non-trained controls. CA1 tissue was then collected from rats in each group, and tissue lysates were immunoprecipitated with an anti-galectin-3 antibody and immunoblotted with an anti-p-Ser antibody. These experiments revealed a candidate p-Ser galectin-3 band of the appropriate molecular weight that was dramatically decreased by fear conditioning training (Figure 6A). Fear-conditioning training also decreased galectin-3 levels in tissue lysates (Figure 6A, lower panel). Because the sequence of rat brain galectin-3 lacks a Ser-12 residue (NCBI database), we examined the role of Ser-6 phosphorylation of galectin-3 in fear memory. Animals were divided to three groups and their CA1 areas were transfected with Flag-vector, Flag-galectin-3WT, or Flag-galectin-3S6A (non-phosphorylatable Ser-6 mutant), after which they were subjected to contextual fear-conditioning learning and retention tests. These experiments revealed that immediate freezing responses were similar among the three groups. Overexpression of Flag-galectin-3WT consistently impaired fear retention compared with the control group, but fear memory returned to normal in rats overexpressing Flag-galectin-3S6A (Figure 6B). Plasmid transfection and expression were confirmed by immunoprecipitation and immunoblotting using an anti-Flag antibody (Figure 6B, lower panel).

In the final set of experiments, we further examined the role of galectin-3 phosphorylation at Ser-6 in fear memory formation. Rats were randomly divided into three groups, and their CA1 areas were transfected with Flag-vector, Flag-galectin-3WT or Flag-galectin-3S6D (a p-Ser-6–mimicking mutant), after which they were subjected to contextual fear-conditioning learning and retention tests. These experiments revealed that immediate freezing responses were similar among the three groups of rats. Animals transfected with Flag-galectin-3 WT consistently showed impaired fear retention compared with the control group, but animals transfected with Flag-galectin-3S6D showed worse memory retention compared with animals transfected with Flag-galectin-3WT (Figure 6C). Plasmid transfection and expression were confirmed by immunoprecipitation and immunoblotting using an anti-Flag antibody (Figure 6C, lower panel).

## Discussion

Our results demonstrate that galectin-3 negatively regulates memory formation through inhibition of integrin α3-mediated signaling and phosphorylation of galectin-3 at Ser-6. These results are consistent with reports that integrin and integrin-associated protein facilitate LTP and memory performance in rats (Staubli et al., 1990; Huang et al., 1998). They are also congruent with the finding that galectin-3 levels are increased in the serum of Alzheimer’s disease patients (Wang et al., 2015). Further support comes from the observations that a lack of galectin-3 facilitates motor function recovery after spinal cord injury (Mostacada et al., 2015), and that galectin-3 contributes to hypoxic-ischemia injury and ischemia-induced neuronal death (Doverhag et al., 2010; Satoh et al., 2011), findings that suggest a role for galectin-3 in negative regulation of neuronal plasticity. However, these latter findings probably reflect the role of galectin-3 in microglia and its interaction with neurons. Our results are also consistent with a report identifying α3β1 integrin as a major binding partner for galectin-3 (Saravanan et al., 2009). In addition to binding integrin, galectin-3 may also bind to other β-galactoside-conjugated ECM proteins and negatively regulate memory through inhibition of signaling mediated by these ECM proteins. For example, galectin-3 was shown to also bind N-cadherin, laminin, fibronectin, and collagen IV (Fortuna-Costa et al., 2014). These results are congruent with the idea that galectins are modulators of cell adhesion (Hughes, 2001). On the other hand, our results are incompatible with a report that phosphorylated galectin-3 promotes axonal branching in cultured hippocampal neurons (Díez-Revuelta et al., 2010). It is possible that interaction of galectin-3 with different cell adhesion molecules might yield different results, because in this latter study, galectin-3 was found to interact with L1. The glutamate NMDAR also plays a key role in mammalian learning and memory formation. But our demonstration that galectin-3 does not interact with the NR1 subunit of NMDAR suggests that the negative regulatory effects of galectin-3 on memory are not mediated by inhibition of NMDAR-mediated signaling. Because galectin-3 inhibits FAK phosphorylation, these results together are consistent with a report that FAK phosphorylation is NMDAR-independent (Yang et al., 2003). Moreover, both fear-conditioning training and NMDA administration down-regulated the expression of galectin-3. How this latter NMDAR-mediated signaling pathway negatively regulates galectin-3 expression warrants further investigation. On the other hand, NMDA is also cytotoxic towards neurons, but this toxicity develops over a much longer time frame (2–3 days after NMDA injection; Kimonides et al., 1998; Harada et al., 1999).

Galectin-3, which is present in both the cytosol and extracellular space (Barondes, 1984; Cherayil et al., 1989), is believed to be externalized through a non-classical secretion mechanism (Cooper and Barondes, 1990). Our results are consistent with these reports in that the association between galectin-3 and integrin α3 decreased after training, and transfection of the FAK phosphorylation-defective mutant blocked the memory-enhancing effect of galectin-3 siRNA. Collectively, these results suggest an extracellular role of galectin-3 in the negative regulation of memory. One possible explanation for these results is that both training and galectin-3 siRNA decrease the expression of galectin-3, which consequently decreases galectin-3 secretion into the extracellular space and its interaction with integrin. STAT1-mediated signaling is another mechanism that might underlie galectin-3–mediated impairment of memory, given that galectin-3 has been shown to increase STAT1 phosphorylation (Jeon et al., 2010) and we have previously shown that STAT1 phosphorylation impairs memory formation (Tai et al., 2011). Moreover, we found that blockade of galectin-3 phosphorylation enhanced memory, suggesting an intracellular role for galectin-3 in the negative regulation of memory. Because galectin-3 is also phosphorylated by CK2 in addition to CK1 in mice (Kübler et al., 2014), this result is congruent with the finding that CK2 activation impairs spatial memory in rats (Chao et al., 2007). In addition to CK1 and CK2, c-Abl kinase also phosphorylates galectin-3 at several tyrosine residues (Balan et al., 2010). Whether galectin-3 phosphorylation at these residues also modulates memory function is not known. Phosphorylation of galectin-3 has been shown to modulate galectin-3 binding to its ligands (Mazurek et al., 2000). Identification of proteins that interact with galectin-3 in neurons and elucidation of the molecular mechanism underlying galectin-3 phosphorylation-mediated memory impairment will require further investigation.

In the present study, fear-conditioning training and water-maze training both decreased galectin-3 expression by approximately 70% in the punched area, which accounts for ~45% of the total CA1 area. These results suggest that about 30% of total CA1 neurons are activated during training that encode memory. In other studies, we have found approximately a 60% reduction in STAT1 expression and a 150% increase in protein inhibitor of activated STAT1 (PIAS1) expression in a subset of CA1 neurons that were dissected out in the same way after spatial training (Tai et al., 2011; Hsu et al., 2014). However, our result does not exclude the possibility that the CA1 neurons that were not punched out are not involved in learning and memory formation.

In this study, galectin-3–KO mice showed enhanced fear retention compared with WT controls. It is thought that other factors besides galectin-3 may also contribute to retention performance; accordingly, we measured the shock sensitivity, locomotor activity, and the anxiety state of these mice. Our results indicated that WT and galectin-3–KO mice showed similar motor scores and number of vocalizations in response to a wide range of foot shock intensities (Supplementary Figure S2). They also showed similar amounts of locomotor activity and comparable movement speed (Supplementary Figure S3). Although the amount of movement by galectin-3–KO mice in the center region of the activity chamber trended higher (Supplementary Figure S3C), this difference did not reach statistical significance, suggesting that anxiety levels are not higher in galectin-3–KO mice than in WT animals.

In this study, we found that training markedly decreased galectin-3 expression, but the extent of fear memory enhancement in galectin-3–KO mice was less dramatic. It is possible that compensatory mechanisms involving other members of the galectin family may exist in galectin-3–KO mice. Because galectin-1 is also expressed in the hippocampus and has been implicated in memory formation (Sakaguchi et al., 2011), we also examined the role of galectin-1 in contextual fear memory. Our results revealed that contextual fear conditioning training also significantly decreased the level of galectin-1 expression in the rat CA1 area (Supplementary Figure S4A). Direct NMDA injection into the rat CA1 area similarly decreased galectin-1 expression (Supplementary Figure S4B). Moreover, knockdown of galectin-1 by transfection of the rat CA1 area with siRNA targeting galectin-1 enhanced fear retention (Supplementary Figure S4C). The transfection and knockdown efficiency of siRNA targeting galectin-1 was confirmed by the marked decrease of galectin-1 expression (Supplementary Figure S4D). These results suggest that one possible candidate galectin that may compensate for the effect of galectin-3 KO in memory formation is galectin-1, which we found also plays a role in negatively regulating memory formation. This speculation may partially explain why galectin-3–KO mice did not exhibit a dramatic enhancement of memory retention. Further support comes from the observations that loss of galectin-1 increases neurogenesis in the subgranular zone in adult mice (Imaizumi et al., 2011) and that galectin-1 causes degeneration of neuronal processes (Plachta et al., 2007), findings that implicate galectin-1 in the negative regulation of neuronal plasticity. However, our results are not consistent with a report that galectin-1–deficient mice show impaired contextual and spatial memory (Sakaguchi et al., 2011). The mechanism underlying the ability of galectin-1 to mediate memory facilitation, revealed in this study, is not known. Examining the effect of galectin-1 siRNA transfection in galectin-3–KO mice on memory retention might help illuminate this issue. But this experiment is difficult to carry out because the animal needs at least 1 week to recover from the surgery associated with siRNA transfection, but the effect of galectin-1 siRNA is not maintained after one week, and repeated siRNA transfection causes dramatic damage to hippocampal neurons. It would probably be also helpful to examine memory retention in galectin-1/galectin-3 double-KO mice. However, there are 15 members of the galectin family (galectin-1–15; Rabinovich and Toscano, 2009), and the distribution of these galectins in the brain and their individual roles in memory performance have not yet been examined. Whether other galectin proteins might also compensate for the effect of galectin-1/galectin-3 double-KO on memory retention is not known and awaits further investigation.

## Conclusion

Galectin-3 promotes inflammatory responses and regulates various cellular functions, but its role in the brain has been much less intensively investigated. In this study, we found that galectin-3 is expressed endogenously in the mouse hippocampal CA1 area and in different regions of the rat brain. Overexpression of galectin-3 impairs fear memory, whereas fear retention, spatial memory, and LTP are enhanced in galectin-3–KO mice. Further, galectin-3 negatively regulates fear memory through inhibition of integrin α3-mediated signaling and through phosphorylation of galectin-3 at Ser-6. Collectively, our findings identify a novel role of galectin-3, showing that galectin-3 negatively regulates hippocampus-dependent memory formation through both extracellular and intracellular mechanisms.

## Author Contributions

Y-CC and EH-YL have designed the research; Y-CC, Y-LM, C-HL, W-LH and S-JC have performed the research; Y-CC, Y-LM and C-HL have analyzed the data; Y-CC and EH-YL have written the article.

## Conflict of Interest Statement

The authors declare that the research was conducted in the absence of any commercial or financial relationships that could be construed as a potential conflict of interest.

## Supplementary Material

FIGURE S1 **(A)** An illustration showing the position and inner diameter of the punch (dotted circle), and the CA1-containing tissue (marked by the oblique lines) that is punched out for biochemical assays in the rat. The lines in white color mark the boundary of the CA1 layer. Scale bar equals 500 μm. **(B)** Immunohistochemistry in WT mice incubated with the FITC-conjugated secondary antibody only. Scale bar equals 200 μm for the upper panel and it equals 100 μm for the lower panel. **(C)** Immunohistochemistry of galectin-3 in galectin-3 KO mice. Scale bar equals 200 μm for the upper panel it equals 100 μm for the lower panel. Arrows in the lower panel of **(B,C)** indicate the same area as shown by arrows in the upper panel of **(B,C)** at a higher magnification.

FIGURE S2 Shock sensitivity measures are similar in WT and galectin-3 KO mice. The WT and galectin-3 KO mice were subjected to foot shock stimulation over a wide range of shock intensities and their **(A)** Motor score (*F*_(1,10)_ = 4.05, *P* > 0.05) and **(B)** Number of vocalizations (*F*_(1,10)_ = 0.15, *P* > 0.05) in response to these shocks are shown. *N* = 6 each group. Data are mean ± SEM.

FIGURE S3 Locomotor activity measures are similar in WT and galectin-3 KO mice. The WT and galectin-3 KO mice were subjected to locomotor activity measures in an activity chamber and the **(A)** Number of crossovers (*t*_(1,10)_ = 0.07, *P* > 0.05), **(B)** Total distance moved (*t*_(1,10)_ = 0.06, *P* > 0.05), **(C)** Distance moved in the center region (*t*_(110)_ = 1.24, *P* > 0.05) and **(D)** Mean speed of movement (*t*_(1,10)_ = 0.6, *P* > 0.05) were recorded. *N* = 6 each group. Data are mean ± SEM.

FIGURE S4 Contextual fear conditioning training and NMDA decrease galectin-1 expression in the rat hippocampus. **(A)** Western blot showing galectin-1 expression and quantitative analysis of galectin-1 expression in the CA1 area of trained and non-trained rats (*t*_(1,10)_ = 76.74, *P* < 0.001). *N* = 6 each group. **(B)** Western blot and quantitative analysis showing the effect of NMDA injection (12.5 mM) on galectin-1 expression in rat CA1 area (*t*_(1,10)_ = 13.54, *P* < 0.001). *N* = 6 each group. **(C)** Effect of galectin-1 siRNA transfection on contextual fear memory in rats (*t*_(1,13)_ = 2.3, *P* < 0.05). *N* = 7 or 8. **(D)** Western blotting showing galectin-1 expression in control siRNA and galectin-1 siRNA-transfected animals. The quantified result is also shown (*t*_(1,13)_ = 26.43, *P* < 0.001). Gal-1: galectin-1. Data are mean ± SEM. **P* < 0.05, ^#^*P* < 0.001.
