# Supplementary figures and images for: Galectin-3 Negatively Regulates Hippocampus-Dependent Memory Formation through Inhibition of Integrin Signaling and Galectin-3 Phosphorylation
Source: Front Mol Neurosci. 2017 Jul 11;10:217. doi: 10.3389/fnmol.2017.00217 (PMC5504160; doi:10.3389/fnmol.2017.00217)

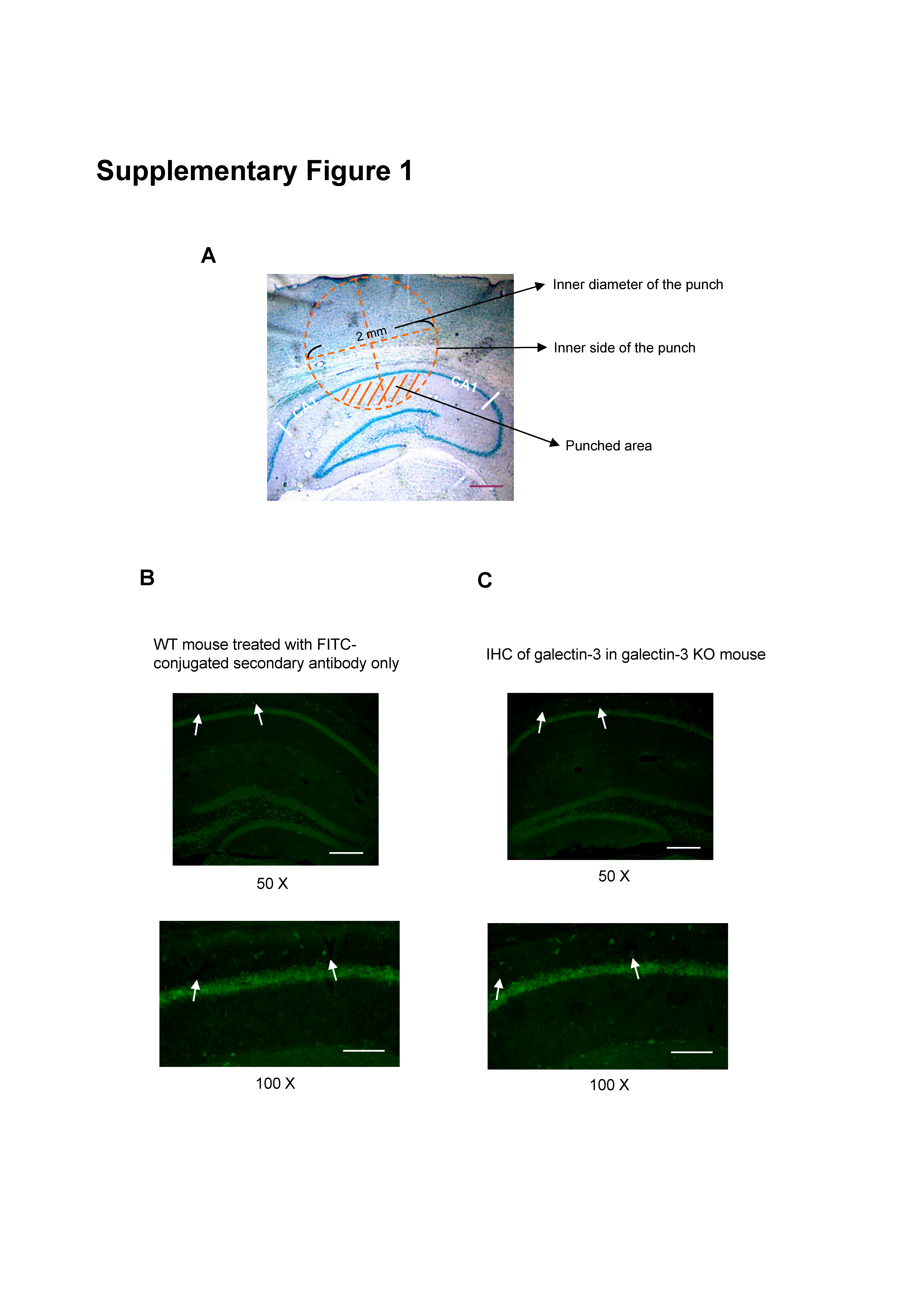

Supplement: FIGURE S1 — (A) An illustration showing the position and inner diameter of the punch (dotted circle), and the CA1-containing tissue (marked by the oblique lines) that is punched out for biochemical assays in the rat. The lines in white color mark the boundary of the CA1 layer. Scale bar equals 500 μm. (B) Immunohistochemistry in WT mice incubated with the FITC-conjugated secondary antibody only. Scale bar equals 200 μm for the upper panel and it equals 100 μm for the lower panel. (C) Immunohistochemistry of galectin-3 in galectin-3 KO mice. Scale bar equals 200 μm for the upper panel it equals 100 μm for the lower panel. Arrows in the lower panel of (B,C) indicate the same area as shown by arrows in the upper panel of (B,C) at a higher magnification. [file Image_1.tiff]
